# Preclinical Development of Bioengineered Allografts Derived from Decellularized Human Diaphragm

**DOI:** 10.3390/biomedicines10040739

**Published:** 2022-03-22

**Authors:** Silvia Barbon, Elena Stocco, Martina Contran, Federico Facchin, Rafael Boscolo-Berto, Silvia Todros, Deborah Sandrin, Filippo Romanato, Piero Pavan, Veronica Macchi, Vincenzo Vindigni, Franco Bassetto, Raffaele De Caro, Andrea Porzionato

**Affiliations:** 1 Section of Human Anatomy, Department of Neuroscience, University of Padova, 35121 Padova, Italy; silvia.barbon@unipd.it (S.B.); elena.stocco@unipd.it (E.S.); martina.contran@unipd.it (M.C.); rafael.boscoloberto@unipd.it (R.B.-B.); veronica.macchi@unipd.it (V.M.); andrea.porzionato@unipd.it (A.P.); 2 Life Lab Program, Consorzio per la Ricerca Sanitaria, 35121 Padova, Italy; federico.facchin@unipd.it (F.F.); sandrin.deborah@gmail.com (D.S.); filippo.romanato@unipd.it (F.R.); vincenzo.vindigni@unipd.it (V.V.); franco.bassetto@unipd.it (F.B.); 3 Foundation for Biology and Regenerative Medicine, Tissue Engineering and Signaling—TES, Onlus, 35030 Padova, Italy; 4 Plastic Surgery Unit, Department of Neuroscience, University of Padova, 35121 Padova, Italy; 5 Department of Industrial Engineering, Centre for Mechanics of Biological Materials, University of Padova, Via Venezia 1, 35131 Padova, Italy; silvia.todros@unipd.it (S.T.); piero.pavan@unipd.it (P.P.); 6 Department of Physics and Astronomy “G. Galilei”, University of Padova, 35131 Padova, Italy

**Keywords:** skeletal muscle, human diaphragm, decellularization, extracellular matrix, tissue engineering

## Abstract

Volumetric muscle loss (VML) is the traumatic/surgical loss of skeletal muscle, causing aesthetic damage and functional impairment. Suboptimal current surgical treatments are driving research towards the development of optimised regenerative therapies. The grafting of bioengineered scaffolds derived from decellularized skeletal muscle may be a valid option to promote structural and functional healing. In this work, a cellular human diaphragm was considered as a scaffold material for VML treatment. Decellularization occurred through four detergent-enzymatic protocols involving (1) sodium dodecyl sulfate (SDS), (2) SDS + Tergitol^TM^, (3) sodium deoxycholate, and (4) Tergitol^TM^. After decellularization, cells, DNA (≤50 ng/mg of tissue), and muscle fibres were efficiently removed, with the preservation of collagen/elastin and 60%–70% of the glycosaminoglycan component. The detergent-enzymatic treatments did not affect the expression of specific extracellular matrix markers (Collagen I and IV, Laminin), while causing the loss of HLA-DR expression to produce non-immunogenic grafts. Adipose-derived stem cells grown by indirect co-culture with decellularized samples maintained 80%–90% viability, demonstrating the biosafety of the scaffolds. Overall, the tested protocols were quite equivalent, with the patches treated by SDS + Tergitol^TM^ showing better collagen preservation. After subcutaneous implant in Balb/c mice, these acellular diaphragmatic grafts did not elicit a severe immune reaction, integrating with the host tissue.

## 1. Introduction

Volumetric muscle loss (VML) is defined as the loss of at least 20% of a given muscle [1,2], which is caused by major trauma or tumour excision [3,4,5], with consequent irrecoverable functional impairment, as well as aesthetic damage [6]. In the orthopaedic field, skeletal muscle injuries can deeply influence the therapeutic outcome of fractures, as well as lead to functional alterations of muscles and limbs, resulting in disabilities [6]. Although these injuries are not commonly life threatening, they profoundly impact the quality of life of patients, since impaired muscle function can alter their body movements and lead to instability, preventing them from carrying out daily activities and being independent [7,8]. For this reason, defining efficient therapeutic strategies to promote the functional regeneration of skeletal muscle is of great clinical and scientific interest.

Regarding the conservative treatment, traditional rehabilitative therapies (i.e., physical therapy) are the mainstay of care for VML injury but have demonstrated limited benefit toward functional recovery in available clinical reports [9].

On the other hand, current surgical treatments of VML injuries are limited to scar tissue debridement and placement of autologous muscle grafts and flaps around the site of tissue defects [10,11]. Unfortunately, these two clinical options are still inefficient, being affecting by many criticisms such as the unavailability of tissues [12], the morbidity of the donor site [4,13], and the occurrence of multiple side effects (i.e., infections, necrosis) [14,15]. Furthermore, these procedures are not always sufficient to adequate restoration of aesthetic and functional features of the affected organ [16].

Given the limits of current therapies, there exists an urgent need for novel treatment options that can promote the innate ability of skeletal muscle to regenerate and restore function following severe trauma in VML patients.

Tissue Engineering (TE) approaches may represent the most promising option to fill this clinical gap in the field of skeletal muscle reconstructive surgery. Initially, strategies that aimed to support muscle reconstruction were focused on cellular approaches, which mainly involved the transplant of exogenous cells with myogenic potential. However, these techniques have proved to be limited by low engraftment in the host tissue and lack of a long-lasting effect [17].

Alternative approaches rely on the use of biological scaffolds, obtained from natural or synthetic materials (e.g., PLGA), as delivery platforms for exogenous myogenic progenitor cells [2,17]. Furthermore, acellular scaffolds derived from tissue extracellular matrix (ECM) have been suggested as biological niches to drive the recruitment and differentiation of endogenous myogenic progenitors, obviating the need to administer exogenous cells [4,18]. Extracellular matrix structure and biochemistry are fundamental elements for muscle regeneration, being capable to restore specific muscular function in VML injuries. Muscle ECM is rich in laminin, fibronectin, collagens, proteoglycans, and growth factors, which orchestrate myoblast differentiation and muscle fibre formation [19,20]. Biomaterials prepared through the decellularization of soft tissues typically retain these ECM constituents, turning out to be promising scaffolds for VML injury repair [18,21,22]. Several decellularized allogenic and xenogenic matrices have already been tested in animal models [23,24] and are also available for clinical use [25], being mainly produced from thin tissues such as the skin [24], small intestine submucosa (SIS) [23,24,25], and bladder [24,25]. Those thin-walled tissues do not fully resemble peculiar properties of skeletal muscle, such as alignment and muscle-specific biochemistry. For this reason, decellularized matrices derived from the skeletal muscle itself seem to be the best choice to efficiently resemble the morphology and biochemical composition of the damaged tissue, supporting both structural and functional healing. To date, skeletal muscle collected from mouse, rat, rabbit, and pig has been largely investigated both in vitro and pre-clinically to develop biocompatible xenografts through decellularization treatment [18]. On the contrary, skeletal muscle of human origin is still poorly considered for the fabrication of tissue-engineered allografts for VML therapy [26]. In particular, human diaphragmatic skeletal muscle may represent a favourable tissue source to obtain biological scaffolds with myogenic potential. The decellularization of mouse [27,28,29,30], rat [31,32,33,34,35,36,37,38], bovine [39,40], and pig [41,42,43] diaphragm was previously reported to fabricate biological prosthesis mainly intended for orthotopic implants in the regeneration of diaphragmatic defects [27,28,32], including neonatal congenital hernia [29,30,31,34,43]. Regarding different clinical targets, the development of decellularized diaphragmatic muscle was also conceptualised for chronic wounds treatment [41], abdominal wall repair [39,40], and anterior cruciate ligament reconstruction [42]. Interestingly, Davari and collaborators firstly reported the preclinical investigation of decellularized human hemidiaphragm for large defect reconstruction of the muscle [44].

Despite progress in the field, scant evidence exists about human diaphragm decellularization for the development of acellular scaffolds for VML therapy. The non-orthotopic use of the xenogenic diaphragmatic matrix for skeletal muscle regeneration has already been shown to give successful outcomes in pre-clinical models of abdominal wall damage [39,40]. Thus, the human diaphragm could be conceived as an additional tissue source for skeletal muscle regeneration in VML patients, to broaden the range and availability of biological prosthesis with ideal regenerative cues. For example, severe loss of abdominal wall domain may require the preparation of muscular grafts of sizeable dimensions, which could be better accomplished by the engineering of a large muscle such as the diaphragm. Furthermore, the diaphragm is a thin, laminar muscle with a different three-dimensional morphology with respect to limb muscles, which could make its use more advantageous when diaphragmatic or abdominal muscle damage needs to be treated. Nevertheless, according to the current literature on the topic, the human diaphragm is still under-investigated for muscle tissue engineering purposes.

Based on the above considerations, this work focused on standardizing a protocol for human diaphragm decellularization by comparing the use of different detergents. Acellular diaphragmatic patches were, then, in vitro characterised to assess the quality of the acellular ECM, while their biocompatibility was verified by in vivo subcutaneous implant into mouse models.

## 2. Materials and Methods

### 2.1. Sample Collection

Human diaphragms (*n* = 6) were harvested from cadaver donors enrolled by the Body Donation Program of the Section of Human Anatomy, University of Padova [45,46,47,48], in compliance with European, Italian, and regional guidelines [49,50]. Donors (age range: 69–73 years; mean age: 71.0 ± 1.5 years) did not have any history of local or systemic pathologies that cause morphological/functional changes in skeletal musculature. In parallel, respiratory pathologies, inflammatory disorders, and muscular tumour co-morbidities, as well as evident signs of diaphragm sarcopenia on dissection were also excluded.

After en bloc sampling of the whole diaphragmatic muscle, patches of about 2.5 × 2.5 cm^2^ were prepared and frozen at −20 °C before starting the decellularization treatment.

### 2.2. Decellularization of Human Diaphragmatic Muscle

All the reagents used for the decellularization of diaphragmatic patches were purchased by Merck Life Science (Darmstadt, Germany). Samples were thawed at room temperature (RT) and manipulated under sterile conditions to avoid contamination by microorganisms. Patches were then cleaned from the surrounding tissue debris, in particular adipose tissue, and extensively washed with 3% antibiotic solution in phosphate buffer saline (PBS), in order to remove any contaminants. Further washes were then carried out with antibiotic solution in decreasing concentration, until the last washing in PBS only to eliminate any residual antibiotic.

Four detergent-enzymatic protocols were tested by comparing the following detergents for human diaphragmatic muscle decellularization (1) sodium dodecyl sulphate (SDS), (2) SDS + Tergitol^TM^, (3) sodium deoxycholate (SDC), and (4) Tergitol^TM^. Protocol details are reported in Table 1.

### 2.3. Characterisation of Decellularized Diaphragmatic Patches

#### 2.3.1. Quantification of Residual DNA

To assess the effective removal of immunogenic material, residual DNA was extracted from the decellularized samples by the DNeasy Blood and Tissue Kit (Qiagen, Düsseldorf, Germany) according to the manufacturer’s protocol. Briefly, samples (10 mg) were lysed with Proteinase K (Merck Life Science) at 56 °C overnight and lysates were loaded onto the DNeasy Mini spin columns for selective purification of total DNA. Eluted DNA finally underwent fluorometric quantification by Qubit 4 fluorometer and kit (ThermoFisher Scientific, Waltham, MA, USA).

#### 2.3.2. Histological Investigations

After decellularization process, native and treated samples were fixed with 10% formalin, paraffin-embedded, cut into 5 μm thick sections and assessed by histological analyses according to routine protocols. In particular, haematoxylin and eosin staining was performed to verify the effective removal of cell nuclei and muscle fibres and Azan-Mallory staining was performed to assess the loss of myofibrillar component and the maintenance of collagen component. In parallel, Weigert Van Gieson staining aimed at demonstrating the persistence of elastic fibres, while Masson’s trichrome staining detected preserved collagen, confirming the absence of muscle fibres. For all the histological stainings, sections were de-waxed and rehydrated with a series of ethanol (Arco Scientifica S.r.l., Padua, Italy) solutions (99%, 95%, and 70%) and distilled water.

#### 2.3.3. Morphometric Analysis

Quantitative morphometric evaluations of the collagen and elastic constituents in decellularized versus native diaphragm patches were performed on Azan-Mallory- and Weigert Van Gieson-stained sections, by using Image J software (version 1.53c, U. S. National Institutes of Health, Bethesda, Rockville, MD, USA) [51] and applying image analysis procedures previously described [4,52]. In particular, the contents of connective tissue were quantified as percentage areas stained in blue (collagen) and purplish (elastin) with Azan-Mallory and Weigert Van Gieson, respectively. Pictures of stained sections were taken considering 8 different fields/section for each experimental group. Images were acquired in bright field at a 10× magnification and saved as TIFF files. Blue and purplish areas were identified by displaying histograms of the distribution of hue, saturation and brightness and setting adequate thresholds for each of these parameters. Specific hue, saturation and brightness ranges were, respectively, 128–200, 0–255, and 0–224 for blue colour (collagen) and 127–225, 0–255, and 0–185 for purplish colour (elastic fibres). Colour ranges corresponding to the collagen/elastic fibres were manually selected and maintained for all the morphometric analyses. Moreover, these colour ranges were converted into black and all the other colours into white. For easier evaluation, the white and black colours were inverted. On the processed images, the white-coloured areas corresponding to collagen/elastic fibres were selected and automatically measured. Thus, results are presented as percentage areas stained in blue/purplish out of the total area of the acquisition field.

#### 2.3.4. Immunohistochemical Study

The adequate preservation of tissue-specific ECM proteins after the decellularization treatments was assessed by the immunolocalisation of Collagen I and IV, as well as Laminin. At the same time, non-immunogenicity of the acellular scaffolds was verified by the investigation of MHC class II (HLA-DR) antigens. Immunohistochemical reactions were carried out by Dako Autostainer/Autostainer Plus (Dako, Milan, Italy) with the following antibodies diluted in PBS: anti-Collagen I (polyclonal rabbit anti-COL1A1, sc-28657, Santa Cruz Biotechnology, Dallas, TX, USA) (1:500); anti-Collagen IV (monoclonal mouse anti-COL4A3, sc-52317, Santa Cruz Biotechnology) (1:100); anti-Laminin (polyclonal rabbit anti-LAM, L9393, Merck Life Science) (1:200); anti-HLA-DR (monoclonal mouse anti-HLA-DR antigens, M0746, Dako) (1:50). Except for Laminin, epitope retrieval was performed with 10 mM sodium citrate buffer, pH 6.0 (for Collagen I and HLA-DR) or pH 9.0 (for Collagen IV), at 90 °C for 10 min. Sections were then incubated with peroxidase-blocking serum (EnVision FLEX Peroxidase-Blocking Reagent; Dako) for 5 min in order to avoid unspecific binding before incubation for 1 h at room temperature (RT) with the above primary antibodies. Specific binding of the primary antibodies was revealed by incubation with the secondary antibodies (EnVision FLEX Mouse-Linker and EnVision FLEX Rabbit-Linker; Dako) for 15 min and EnVision FLEX/HRP polymer for 20 min. Subsequently, 3,3′-diaminobenzidine (EnVision FLEX Substrate Buffer + DAB + Chromogen; Dako) was used in order to highlight the positivity of the reaction. Finally, the sections were counterstained with haematoxylin. Native diaphragmatic samples were used as reference for marker expression, whereas negative controls were prepared by incubating sections without primary antibodies.

#### 2.3.5. Glycosaminoglycans Quantification

Sulphated glycosaminoglycans (GAGs) were quantified into decellularized diaphragmatic patches versus the native tissue by using the Chondrex Inc. Glycosaminoglycans Assay Kit (DBA Italia S.r.l., Milan, Italy). Tissue samples (10 mg) were first processed for GAG solubilisation by digestion in Papain solution at 56 °C overnight. The cationic dye 1,9 dimethylmethylene blue (DMB) was used to label solubilised GAGs and colorimetric reaction was read at 530 nm by using the Microplate auto reader VICTOR3™ (PerkinElmer, Waltham, MA, USA). Together with diaphragmatic samples, chondroitin sulphate was analysed as a standard for GAG quantification into the specimens.

### 2.4. Second Harmonic Generation Microscopy

Second Harmonic Generation (SHG) imaging was performed on paraffin-embedded label-free tissue sections derived from decellularized diaphragmatic patches in comparison with the native sample by using a custom developed multiphoton microscope, previously described by Filippi et al. [53]. Briefly, an incident wavelength of 800 nm was adopted to detect the collagen’s SHG signal at 400 nm and the AutoF signal at 525 nm on two different photodetectors (GaAsP PMT with 395/25 nm bandpass filter and GaAsP PMT with 525/40 nm bandpass filter, respectively). The images were acquired at a fixed magnification through the Olympus 25× water immersion objective with 1.05 numerical aperture (1024 × 1024 pixels), averaged over 70 consecutive frames, with a pixel dwell time of 0.14 μs and a pixel width of 0.8 μm. For quantitative measurements, the RAW uncompressed images were analysed by using Image-J software. Coherency (C) was calculated for collagen and elastin to verify the local dominant orientation of the images using OrientationJ, an ImageJ plugin [54]. The estimated parameter is bounded between 0 and 1, indicating respectively the absence (isotropy) and the presence (anisotropy) of dominant orientation. A graphic representation of the coherency that shows organisation and distribution of the fibres is given by Fast Fourier Transform (FFT) analysis. The transform-based texture analysis techniques convert the image into a new form using the spatial frequency properties of the pixel intensity variations allowing the extraction of textural characteristics from the image. Indeed, highly oriented fibre in a single direction shows an elliptic shape; differently, a circular shape represents fibre spread in all directions [55,56].

### 2.5. Tensile Testing

Patches of native and decellularized diaphragmatic tissue from a single donor were cut into rectangular samples in longitudinal (L) and transversal (T) directions with respect to the muscular fibres. A gauge length of 15 mm and nominal width of 5 mm were selected to ensure a mean aspect ratio (length/width) of about 3, in accordance with other test protocols [57,58]. At least three samples were obtained for each test direction and for each treatment. The effective width and thickness of the samples were measured by means of ImageJ from top and side view pictures of each specimen. Tissue samples were glued between pieces of balsa wood at either end to prevent slippage during testing, then clamped by the grips and hydrated by dropping PBS regularly. Mechanical tests were carried out with Bose ElectroForce^®^ Planar Biaxial Test Bench instrument (TA Instruments, New Castle, DE, USA) under displacement control with precision of ± 0.001 mm and adopting a load cell of 22 N with a precision of ±0.02 N. Uniaxial tensile tests consisted of five loading–unloading cycles up to a maximum nominal strain of 20%, at a constant strain rate of 1% s^−1^. Nominal strain ε was determined as the ratio between the displacement of the grips and the initial gauge length of the sample, while the nominal stress P was calculated as the ratio between the force, measured by the load cell, and the initial transversal area of the sample. The secant elastic modulus E_s_ was calculated from the last loading cycle of each sample, as the slope of the straight line drawn from the origin of the stress–strain diagram and intersecting experimental data at 20% strain.

### 2.6. In Vitro Cytotoxicity

#### 2.6.1. Indirect Co-Culture with Adipose-Derived Mesenchymal Stem Cells

Possible release of toxic chemical remnants from decellularized diaphragmatic patches was verified by preparing indirect co-cultures of human adipose-derive mesenchymal stem cells (Ad-MSCs) (Cell Line Service, Eppelheim, Germany) with decellularized diaphragmatic samples by means of cell culture insert membranes. First of all, acellular diaphragmatic scaffolds were sterilised by treatment with 2% penicillin/streptomycin solution (Merck Life Science) for 72 h and regular washes in sterile water for other 72 h. In parallel, Ad-MSCs were first seeded on 24-well plates (5000 cells/cm^2^) into proliferation medium consisting of Mesenchymal Stem Cells Expansion Medium (Cellular Engineering Technologies, Coralville, IA, USA) + 10% Foetal Bovine Serum (Merck Life Science) and 1% penicillin/streptomycin solution (Merck Life Science). After having let cells to adhere and growth for 24 h, indirect contact co-cultures were prepared by adding an insert porous membrane (pore size 8 µm) into each seeded well and positioning a discoidal tissue sample (diameter: 8 mm) onto the membrane. Co-cultures were grown at 37 °C, 5% CO_2_ and 95% humidity for 72 h. In parallel, the positive (cytotoxic) control was prepared by incubating Ad-MSCs in culture medium added with 50% DMSO, whereas the negative control was represented by untreated cultures.

#### 2.6.2. MTT Assay

At the end of the incubation period, the effect of indirect contact co-culture on Ad-MSC viability was assessed by treating cells with 0.5 mg/mL MTT for 4 h and then dissolving the resulting formazan precipitates by 2-propanol acid (0.04 M HCl in 2-propanol). After 15 min under agitation to favour formazan solubilisation, optical density of samples was measured at 570 nm with the Microplate auto reader VICTOR3™ (PerkinElmer). Results of the cytotoxicity test were expressed as percentages of viable and metabolically active cells in treated groups, in comparison with the untreated control, which was set as 100% cell viability.

### 2.7. Cell Seeding on Acellular Diaphragmatic Scaffolds

To test scaffold ability in sustaining cell adhesion and growth, Ad-MSCs at passage 3 were seeded on 4 mm diameter acellular diaphragmatic disks derived from the decellularized patches. Before cell seeding, samples were sterilised as described in Section 2.6.1 and then subjected to microbiological test by culture in basal medium with 10% FBS at 37 °C, 5% CO_2_ and 95% humidity. In the absence of bacterial or fungal contamination, the diaphragmatic disks were placed in a 96-well plate and pre-treated with Ad-MSC proliferation medium at 37 °C overnight to promote cell adhesion. Subsequently, Ad-MSCs were seeded at a density of 10,000 cells/scaffold in 200 µL of proliferation medium. After 7 days of culture onto the scaffolds, cell viability was assessed by MTT assay, as already described in Section 2.6.2. Results of cell seeding were expressed as number of cells detected on scaffolds after having prepared an MTT standard curve. The number of 1000, 5000, 10,000, 20,000, and 100,000 Ad-MSCs/well were seeded in 96-well plates and let adhere for 12 h. Cell viability was then measured by MTT assay, obtaining optical density values to associate to each point of the curve. To quantify cells grown on seeded scaffolds, optical density values measured for each sample were plotted on the standard curve, gaining the correspondent cell number.

In parallel, the ultrastructure of seeded disk surfaces was investigated by scanning electron microscopy (SEM).

#### 2.7.1. Ultrastructural Investigation by Scanning Electron Microscopy

To analyse their superficial ultrastructure, diaphragmatic tissue specimens were fixed with 2.5% glutaraldehyde in 0.2 M phosphate buffer (pH 7.4) for 24 h, washed 4–5 times with phosphate buffer and gradually dehydrated by successive immersion in increasing concentrations of ethanol for 15 min each. After tissues were critical-point dried and coated with an 8 nm gold layer, specimen observation and micrograph acquisition were carried out by using the tungsten thermionic emission SEM system JSM-6490 (Jeol USA, Peabody, MA, USA).

### 2.8. In Vivo Biocompatibility

#### 2.8.1. Subcutaneous Implant

For in vivo biocompatibility study, a biopsy punch was used to prepare discoidal samples (8 mm diameter; 2–3 mm thickness) starting from the diaphragmatic patches decellularized according to Protocol n. 2. Before implant, acellular scaffolds were sterilised by 2% penicillin/streptomycin solution in PBS and exposure to UV light for 30 min.

Six female twelve-week-old mice (mean weight 20.8 ± 1.6 g) were implanted subcutaneously with 1 diaphragmatic scaffold each. After administrating gas anaesthesia (isoflurane/oxygen), animal dorsal cutis was shaved and disinfected with Betadine^®^ (Bayer, Leverkusen, Germany) and a median 10 mm lumbotomy incision was executed by using a No. 10 surgical blade (Becton-Dickinson, Franklin Lakes, NJ, USA) to create a subcutaneous pouch. Diaphragmatic scaffolds were inserted into the pouch and anchored to the latissimus dorsi muscle by using Tycron 4/0 sutures in order to prevent graft displacement. Finally, the skin was stitched by absorbable Novosyn 4/0 sutures. The animals were administered antibiotic and anti-inflammatory therapy for 5 days after surgery and were monitored during the whole recovery period. Euthanasia was performed at postoperative day 14, and diaphragmatic grafts were excised together with the surrounding tissues to perform SEM analysis as described in Section 2.7.1, as well as histological and immunohistochemical investigations.

#### 2.8.2. Histological and Immunohistochemical Analyses

The microscopic morphology of diaphragmatic grafts and surrounding tissues after 14 days of subcutaneous implant was investigated by histological staining with haematoxylin and eosin. Formalin-fixed, paraffin-embedded sections of explants were prepared as previously described. In parallel, the immunolocalization of the lympho-monocytic fraction into the tissue explants was performed by using the following primary antibodies: anti-CD3 (polyclonal rabbit anti-CD3, A0452, Dako) (1:500) and anti-F4/80 (polyclonal rabbit anti-F4/80 (M-17)-R, sc-26643-R, Santa Cruz Biotechnology) (1:1000) to label lymphocytes and monocytes/macrophages, respectively. At the same time, any angiogenic/myogenic infiltration was assessed by labelling endothelial and muscular cells by the primary antibodies anti-Vascular Endothelial Growth Factor (VEGF) (monoclonal mouse anti-VEGF, C-1, sc-7269, Santa Cruz Biotechnology) (1:300) and anti-myosin (monoclonal mouse anti-slow muscle myosin, MAB1628, Merck) (1:1500), respectively. Epitope retrieval (for CD3, F4/80, and VEGF), blocking of unspecific binding sites, and revealing of primary antibody specific binding were carried out as described above.

### 2.9. Statistical Analysis

Data are presented as Mean ± standard deviation (SD) of at least three replicates. The one-way analysis of variance (ANOVA) followed by the Tukey post hoc test for multiple comparisons were used to determine any significant differences among the experimental groups. Specifically, differences were considered significant with *p* ≤ 0.05.

## 3. Results

### 3.1. Diaphragm Decellularization

**Figure 1:**
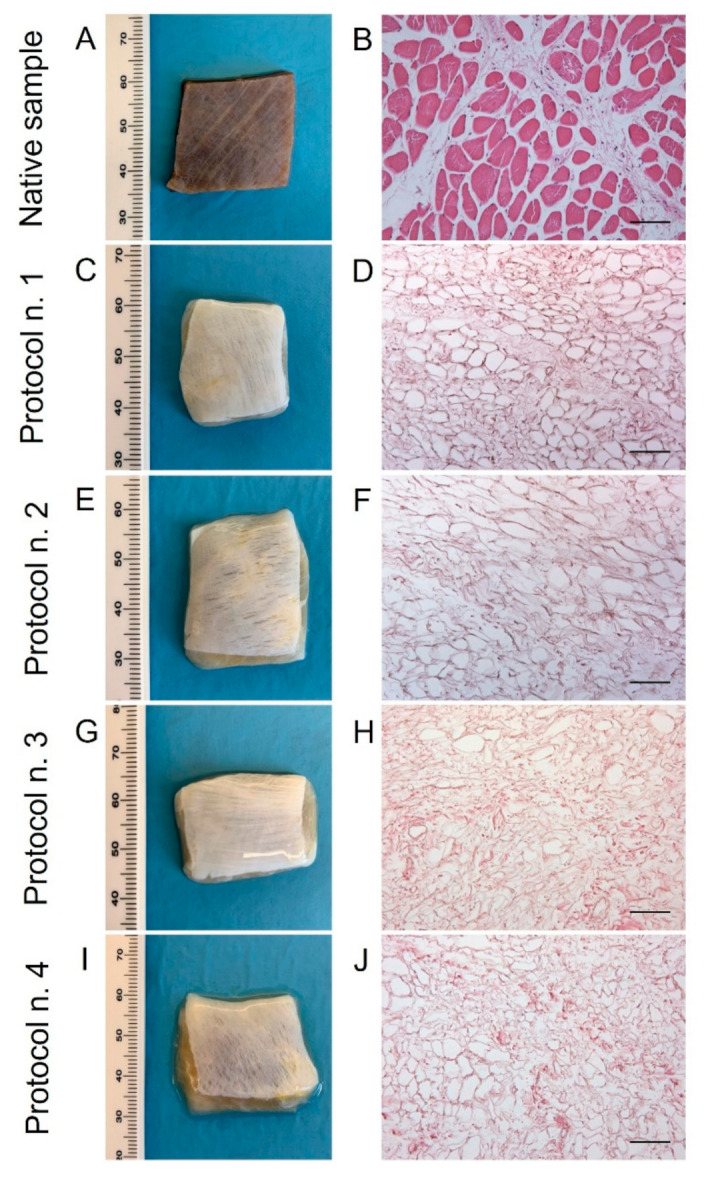
Gross appearance (**A**,**C**,**E**,**G**,**I**) and microscopic morphology (**B**,**D**,**F**,**H**,**J**) of diaphragmatic patches before (**A**,**B**) and after decellularization treatment according to Protocols n. 1 (**C**,**D**), n. 2, (**E**,**F**), n. 3 (**G**,**H**), and n. 4 (**I**,**J**). Macroscopic evaluation highlighted a substantial whitening of tissue samples due to cell and muscle fibre loss, while haematoxylin and eosin staining assessed the efficient removal of cell nuclei and myofibrils into all the decellularized specimens compared to the native diaphragm. Scale bar: 75 µm.

After human diaphragm sampling, tissue preparation, and decellularization were performed under sterile conditions to avoid bacterial/fungi contamination. Following detergent-enzymatic treatment, all the diaphragmatic patches showed to preserve adequate volume and homogeneity when compared to the native control. No tissue ruptures were observed, and treated samples appeared to maintain manipulability for surgical sutures (Figure 1A,C,E,G,I). Preliminary macroscopic evaluation demonstrated that decellularized tissues assumed a translucent quality and underwent a substantial whitening in comparison with the native specimens, suggesting a progressive loss of the cellular component (Figure 1A,C,E,G,I). The consistency of diaphragmatic patches seemed to be altered after decellularization in comparison to the native tissue, with more evident effects produced on the samples treated by long-term incubation in Tergitol^TM^ detergent (Protocol n. 4), which tended to collapse during manipulation.

### 3.2. Histological Investigation

Haematoxylin and eosin staining on native diaphragms demonstrated the typic morphology of striated muscle tissue with transverse striations, large number of nuclei, and intrafibrous connective tissue (Figure 1B). Considering the analysis on treated samples, all the four protocols effectively removed both cellular elements and skeletal muscle fibres, visible cell nuclei or myofibrillar elements being no longer in all the decellularized specimens (Figure 1D,F,H,J).

**Figure 2:**
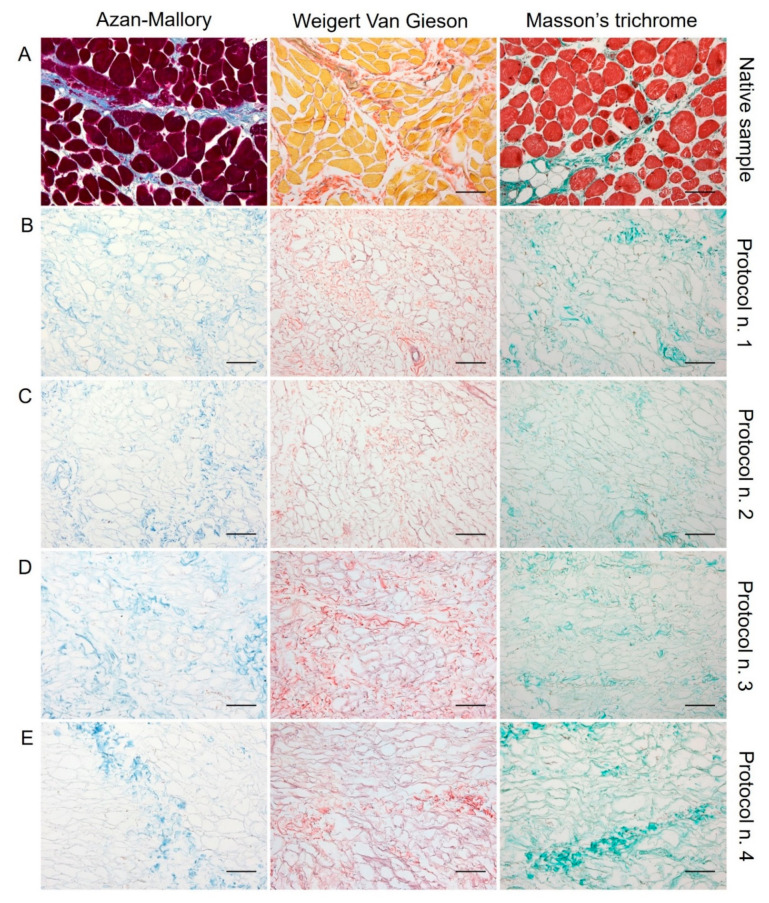
Histological evaluation of diaphragmatic patches before (**A**) and after decellularization treatment according to Protocols n. 1 (**B**), n. 2, (**C**), n. 3 (**D**), and n. 4 (**E**). Overall, Azan-Mallory, Weigert Van Gieson, and Masson’s trichrome stainings demonstrated the removal of cell nuclei and muscle fibres, together with the persistence of ECM components into the decellularized samples (**B**–**E**) versus the native tissue (**A**). Scale bar: 75 µm.

Regarding skeletal muscle ECM, all tested decellularization protocols preserved quite well the protein composition and 3D organisation of the diaphragmatic tissue, with the acellular matrix structure appearing less compact than the native sample. According to Azan-Mallory, Weigert Van Gieson, and Masson’s trichrome staining, muscle fibres (dark red, yellow, and bright red colour, respectively) were no longer detectable in the decellularized samples in comparison with the native tissue (Figure 2). As evidenced by Azan-Mallory and Masson’s trichrome staining, the connective component represented by collagen fibres (blue and green colour, respectively) was not depleted by the decellularization treatments (Figure 2). Additionally, Weigert Van Gieson staining also demonstrated the persistence of elastic fibres (purplish colour) in the ECM network around spaces previously occupied by skeletal muscle fibres (yellow colour) (Figure 2). Overall, decellularization treatments also adequately preserved the vessel components, without compromising the collagen/elastic fibres of the wall, as demonstrated by the fact that the lumens of the vessels were clearly visible in tissue sections. Conversely, nuclei of endothelial and smooth muscle cells were correctly removed.

### 3.3. Immunohistochemical Study

**Figure 3:**
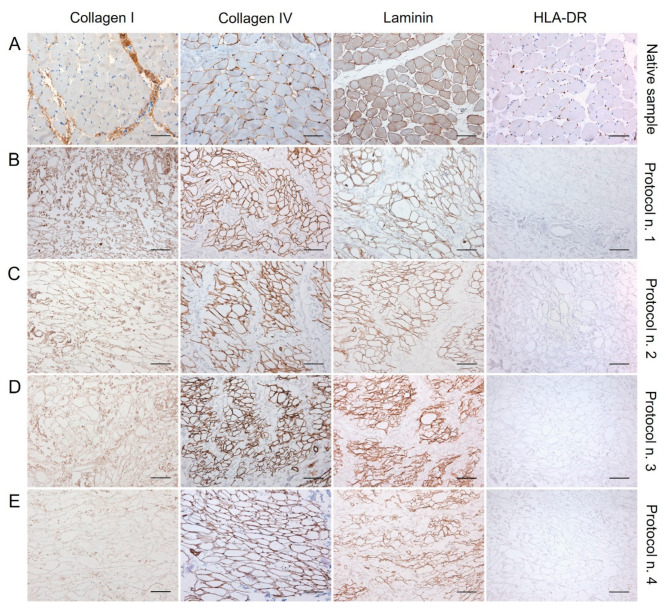
Immunolocalisation of Collagen I, Collagen IV, Laminin, and HLA-DR into diaphragmatic patches before (**A**) and after decellularization treatment according to Protocols n. 1 (**B**), n. 2, (**C**), n. 3 (**D**), and n. 4 (**E**). Specific ECM marker expression was preserved after the decellularization process in comparison with the native diaphragm, while the loss of HLA-DR positivity after detergent-enzymatic treatments indicated the low immunogenicity of acellular grafts. Scale bar: 75 µm.

The examination of specific ECM markers by immunohistochemistry confirmed the presence of Collagen I and IV and Laminin into the decellularized diaphragmatic patches versus the native tissue, with less positive labelling of Collagen I and Laminin into specimens treated with Protocol n. 4 (Figure 3).

Along with specific ECM markers, HLA-DR antigens was also immunolocalised into decellularized and native tissues, to furtherly confirm the loss of immunogenic materials in the acellular graft. Being an MHC class II cell surface receptor, HLA-DR was found to be localised at the cellular level in the native diaphragm, while its expression was not detected in the decellularized samples, confirming low immunogenicity of the scaffolds (Figure 3).

### 3.4. Quantitative Analyses on Acellular Diaphragmatic ECM

**Figure 4:**
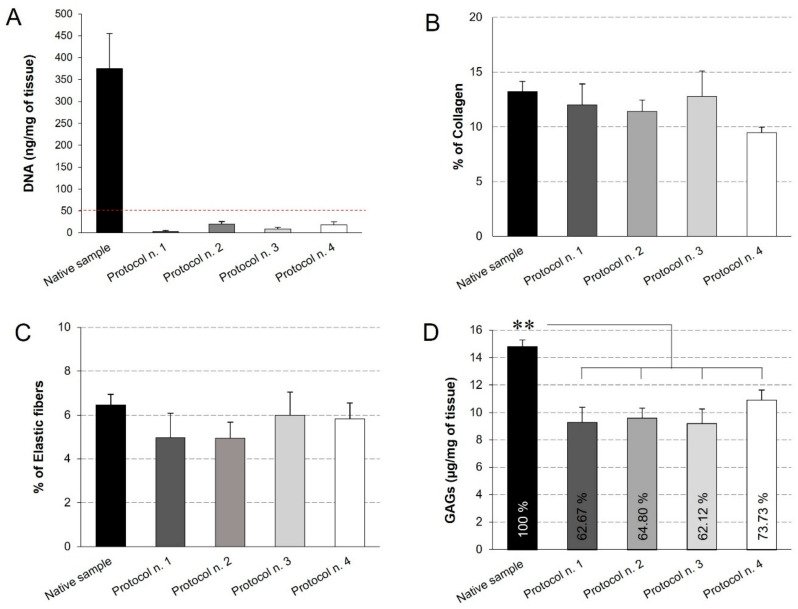
Quantification of residual DNA (**A**), collagen (**B**), elastic fibres (**C**), and glycosaminoglycans (GAGs) (**D**) into decellularized diaphragmatic samples in comparison with the native tissue. Residual DNA was below the threshold of 50 ng/mg of tissue (dashed red line) for all the decellularized samples (**A**). Morphometric analyses detected no significant differences among groups regarding the collagen (**B**) and elastic fibre (**C**) content. The reported percentages correspond to the specific stained area of collagen/elastic fibres out of the total area of the analysis field (**B**,**C**). Finally, all decellularization protocols seemed to significantly reduce the GAG component in comparison with the native tissue, while assuring for the preservation of 60%–70% of glycosaminoglycans (**D**) (**: *p* < 0.01).

Quantitative measures were performed to assess the quality of the decellularized diaphragmatic ECM. First of all, the quantification of residual DNA assessed that all the decellularization protocols led to a significant decrease in the DNA content compared to the native tissue, below the set threshold of 50 ng/mg [59] (Figure 4A). This result is in line with the qualitative analysis by haematoxylin and eosin, which highlighted the loss of cell nuclei in all treated samples. Morphometric analyses on Azan-Mallory- and Weigert Van Gieson-stained sections allowed the residual collagen and elastic fibres within the native and acellular patches to be quantified, pointing out no significant differences among experimental groups and, thus, suggesting that all the detergent-enzymatic protocols were able to retain this ECM components (Figure 4B,C).

Additionally, GAGs quantification by Dimethylmethlyene Blue demonstrated that all the decellularization protocols caused a significant decrease in this ECM component, yet preserving the 60%–70% of native GAG content. At the same time, no statistical difference was found among the four decellularization methods (Figure 4D).

### 3.5. SHG Imaging

**Figure 5:**
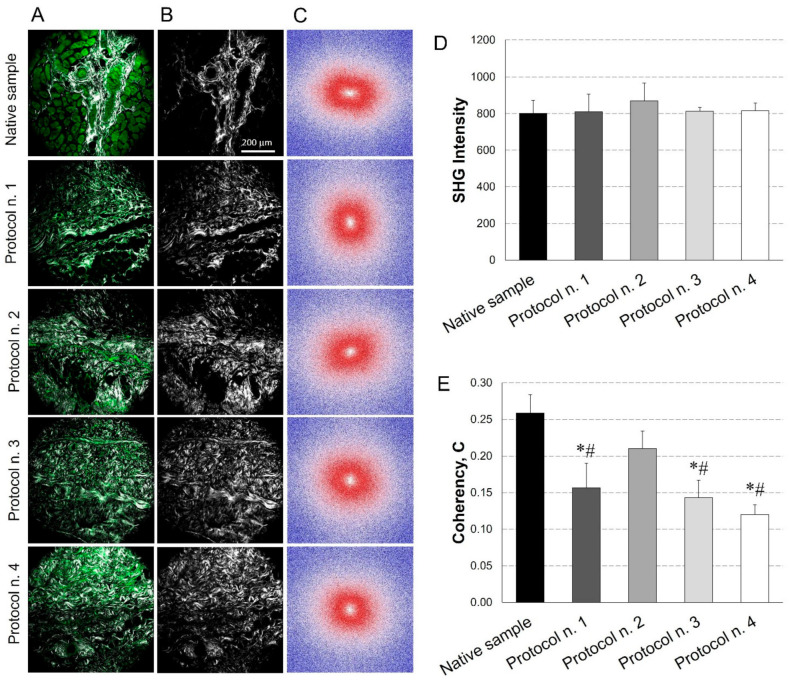
Evaluation of collagen content and distribution into diaphragmatic patches before and after decellularization treatments. Representative images for each sample of the overlay between the AutoFluorescence signal in green and the Second Harmonic Generation (SHG) in grey (**A**); label-free SHG collagen analysis (**B**) and Fast Fourier Transforms (FFTs) showing an ellipsoidal and a spherical fibre orientation for native and decellularized samples, respectively (**C**). SHG intensity evaluation (average values of *n* = 7 images for each sample with the corresponding standard errors): no significant differences were found among experimental groups (**D**). Average values of Coherency (**C**) of collagen, which estimates the local orientation of the fibres. Except for Protocol n. 2, all the decellularization treatments affected collagen fibre orientation (*: *p* < 0.05 vs. Native sample; #: *p* < 0.05 vs. Protocol n. 2) (**E**).

A further characterisation of the acellular diaphragmatic ECM was performed through collagen imaging by SHG microscopy (Figure 5A–C). Mean intensity of the SHG signal for the collagen indicated that its level remained constant after all the decellularization treatments in comparison with the native condition (Figure 5D).

In parallel, FFT analysis provided a measure of collagen fibres orientation. As shown in Figure 5C, in the native tissue, fibres tended to be orientated predominately in one direction, exhibiting anisotropic behaviour (i.e., ellipsoidal shape for FFT); conversely, in decellularized samples, fibres tended to be randomly orientated and demonstrated isotropic behaviour (i.e., spherical shape for FFT), with some area of patches treated with Protocol n. 2 showing similar behaviour as the native tissue. These trends were confirmed by coherency values, which estimated the local orientation of collagen fibres, identifying significant coherency differences after all the decellularization treatments versus the native tissue, except for Protocol n. 2. Additionally, a statistical difference in fibre orientation coherence was observed among Protocol n. 2 and the other decellularization methods (Figure 5E).

### 3.6. Mechanical Behaviour

The stress–strain response of the diaphragmatic tissue during five loading–unloading cycles highlighted a decrease in the stress values with an increasing number of cycles, until stabilisation. From the 1st to the 2nd cycle, a maximum stress reduction in the order of 15% was found, while the percentage reduction progressively decreased below 1% from the 4th to the 5th cycle. This behaviour is typical of dissected biological tissues, which need a preconditioning before reaching a stable mechanical response [57].

**Figure 6:**
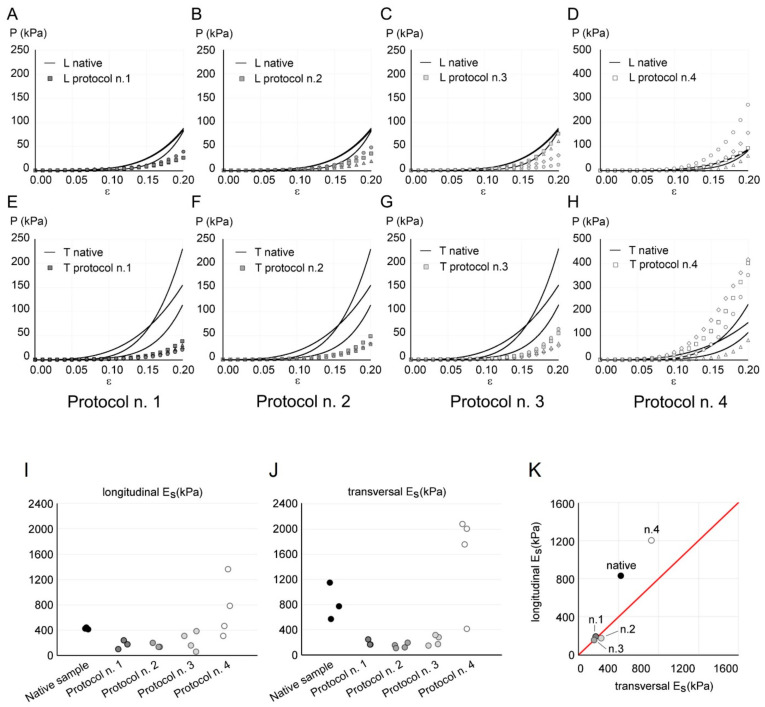
(**A**–**H**) Comparison of the mechanical response of native (continuous line) and decellularized (markers) diaphragmatic tissue with Protocols n. 1 (**A**,**E**), n. 2 (**B**,**F**), n. 3 (**C**,**G**), and n. 4 (**D**,**H**), in longitudinal (indicated as “L” in **A**–**D**) and transversal (indicated as “T” in **E**–**H**) directions. The results are reported as nominal stress P vs. nominal strain ε. Stress values are plotted on a different range for Protocol n. 4, to ensure a clearer data visualisation. (**I**–**K**) Longitudinal (**I**) and transversal (**J**) secant elastic modulus E_s_ calculated from tensile data of each sample of native and decellularized diaphragmatic tissue. (**K**) Mean values of longitudinal E_s_ vs. transversal E_s_ for native tissue and different decellularization protocols. The red line represents isotropy, i.e., a condition in which longitudinal E_s_ and transversal E_s_ are equal.

The experimental results of the fifth cycle of tensile tests at 20% strain are shown in Figure 6A–H, comparing native and decellularized diaphragmatic tissue, according to different protocols. Both native and decellularized samples showed a nonlinear stress–strain behaviour with increasing stiffness with strain level. In the case of native tissue, a certain degree of anisotropy could be noted by comparing the tensile data acquired in longitudinal and transversal directions. This is consistent with previous studies [60], where a lower stiffness of passive diaphragmatic tissue was found along the fibre direction with respect to transversal direction. The same level of anisotropy was not found from stress–strain plots in the case of decellularized tissues. Both in transversal and longitudinal direction, samples obtained via Protocol n. 4 reached higher stress values than native and decellularized samples with all the other protocols.

In order to evaluate the anisotropy and to compare the tissue stiffness before and after decellularization, the elastic secant modulus E_s_ was considered (Figure 6I–K). The ANOVA of E_s_ values with the Kruskal–Wallis test showed a statistically significant difference among the five tested groups (*p*-value = 0.022 in the longitudinal direction, data in Figure 6I; *p*-value = 0.009 in the transversal direction, data in Figure 6J), highlighting a different stiffness among samples obtained with different protocols. Post hoc did not show significant differences among the different protocol, due to the limited size of available dataset. As emphasised in Figure 6K, decellularized samples obtained with Protocols n. 1, n. 2, and n. 3 presented an almost isotropic behaviour; native tissue showed the highest anisotropy degree, which was slightly decreased after decellularization with Protocol n. 4.

### 3.7. Cytocompatibility Studies

Possible scaffold-induced cytotoxicity after decellularization treatment was investigated by indirect co-culture with Ad-MSCs, which are possible candidates for future studies of acellular matrix repopulation. As demonstrated by cell viability assay (Figure 6), no cytotoxic effect was detected on cells co-cultured with decellularized samples for 72 h, with preservation of >80% viability in comparison with the untreated control. No significant differences were observed among experimental groups, except for significantly lower cell viability detected in the cytotoxic control compared to the untreated control and all the co-cultures with decellularized tissues.

**Figure 7:**
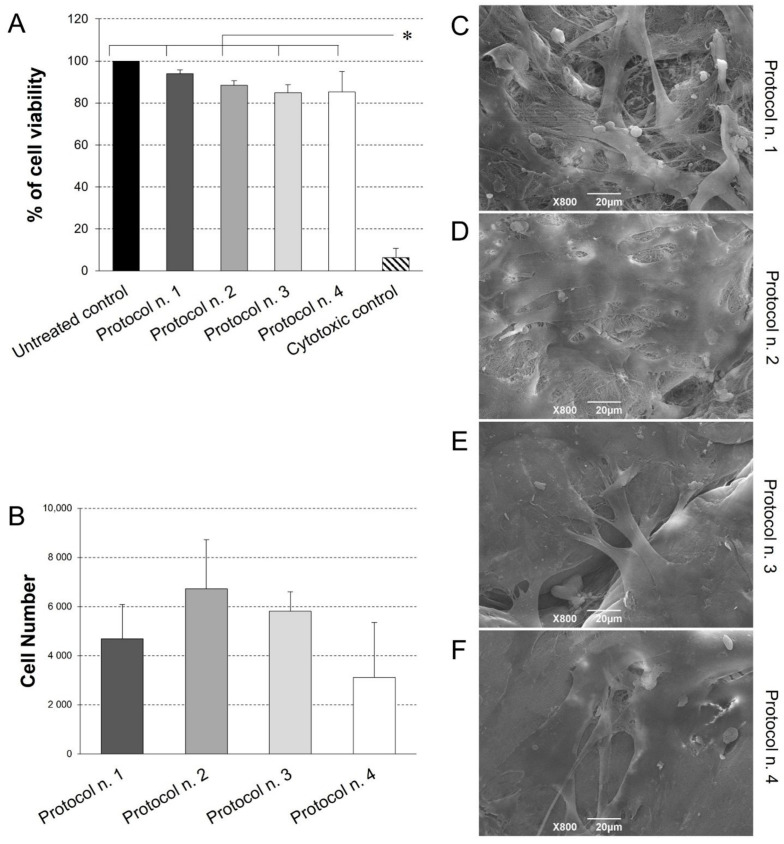
(**A**) Cell viability of adipose-derived mesenchymal stem cells (Ad-MSCs) grown by indirect co-culture with decellularized diaphragmatic samples. Non cytotoxicity of acellular scaffold was confirmed by the preservation of >80% cell viability after indirect contact culture. (* *p* < 0.01). (**B**) Ad-MSC growth on acellular diaphragmatic scaffolds after 7 days of culture in static conditions. Results are presented as number of cells detected on each scaffold by MTT assay. (**C**–**F**) SEM micrographs showing the surface morphology of the seeded acellular diaphragmatic scaffolds, where adherent cells on collagen matrix were visible.

Given the successful outcome of cytotoxicity test, diaphragmatic scaffold recellularization was performed by Ad-MSC seeding for the investigation of cell adhesion and distribution onto the scaffold surface. Viability assay revealed the presence of viable, proliferative cells on acellular matrices treated by the four protocols, with no significant difference among the experimental groups (Figure 7B). Ultrastructural analysis by SEM confirmed these data, showing that Ad-MSC grew on all diaphragmatic patches with their typical fibroblast-like morphology, proliferating as a monolayer of adherent cells on the collagen fibre network (Figure 7C–F).

### 3.8. In Vivo Biocompatibility Study

**Figure 8:**
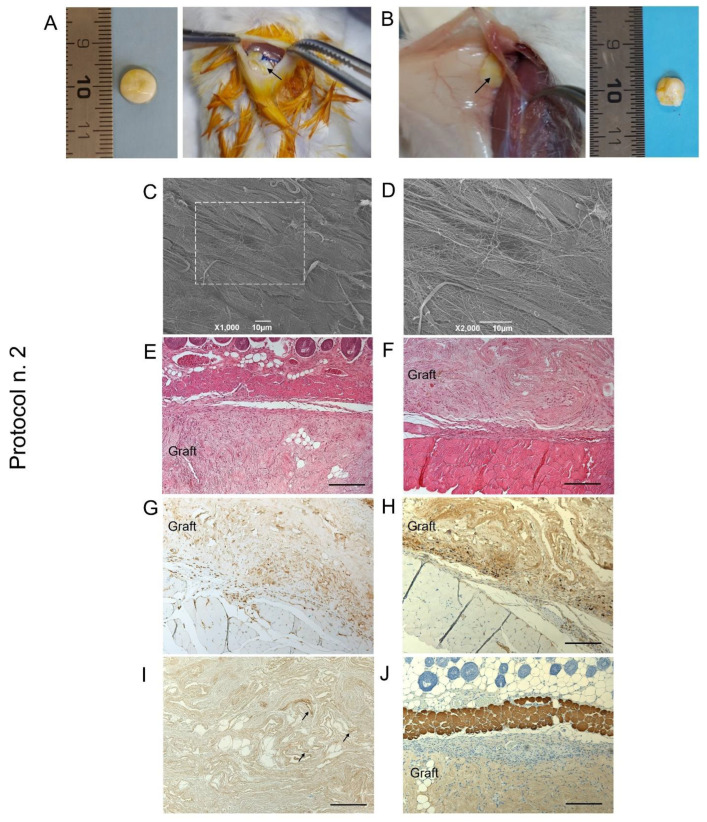
Biocompatibility of decellularized human diaphragm after in vivo implant into BALB/c mice. Discoidal samples with 8 mm diameter were inserted into a dorsal subcutaneous pouch, anchored to the muscle (**A**) and left in place for 14 days before sample excision (**B**). Macroscopic evaluation (**B**), ultrastructural analysis by SEM (**C**,**D**), and histological staining with haematoxylin and eosin at the graft–host interface on the subcutaneous (**E**) and deeper muscular side (**F**) revealed that the graft well integrated with the surrounding tissues, showing no evidence of severe host immune response. The immunolocalization of CD3^+^ (**G**) and F4/80^+^ (**H**) cells at the boundaries between the graft and the host tissue confirmed that only moderate lympho-monocytic infiltration was triggered by the scaffold graft. Finally, positivity to VEGF (black arrows) (**I**) and negativity to myosin (**J**) within the diaphragmatic graft demonstrated that an early angiogenetic, but not myogenetic, process was initiated after 14 days from implantation. Scale bar: 10 µm (**C**,**D**); 200 µm (**E**–**J**).

When decellularized scaffold biocompatibility was assessed by subcutaneous implant experiments in mice, graft material appeared to be suitable for surgical manipulation and exhibited optimal suture retention capacity (Figure 8A,B).

At the time of scaffold retrieval—14 days from surgery—acellular diaphragmatic samples were still well recognisable at the site of implant, with preliminary macroscopic evaluation revealing grade 1 soft tissue adhesion, as well as the formation of a filmy thickness connective layer surrounding the grafts (Figure 8B). Ex vivo analyses on transplanted constructs suggested moderate immunological response by the host after acellular scaffold grafting, with SEM micrographs highlighting the presence of collagen fibres on the sample surface (Figure 8C,D). Histological investigation assessed scaffold biointegration with the surrounding tissues, not showing severe inflammatory infiltrate at the graft–host interface at both the subcutaneous (Figure 8E) and dorsal muscle side (Figure 8F). Evaluating the specific immune response by the treated animals, mild lympho-monocytic invasion was immunolocalised at the graft boundaries, confirming low immunogenicity of the human diaphragmatic constructs (Figure 8G,H). Finally, VEGF-positive cells were detected within the graft (Figure 8I), whereas no myosin expression was recognisable after 14 days of the in vivo implant (Figure 8G).

## 4. Discussion

Bioengineered allografts are arousing more and more interest in the field of Regenerative Medicine. Preserving the ECM of the target tissue, they are made to be immunologically inert thanks to a decellularization treatment that removes the immunogenic components. Consequently, bioengineered allografts do not involve the use of immunosuppressive therapy, offering an ideal and clinically viable alternative to current reconstructive strategies. Human tissues of allogenic origin represent the ideal biomaterials to produce highly biocompatible grafts for implantation into the patient [59]. Such tissues can be obtained during surgical procedures or taken from cadavers. Based on our experience, the creation of a human tissue bank through the promotion of body donation programs, could incentivise the development of innovative tissue regeneration strategies. In this regard, the Body Donation Program of the University of Padova, active at the Section of Human Anatomy—Department of Neuroscience, collects bodies from donors and surgically amputated body parts [45,46,47,48], ensuring the availability of anatomical material that represents a valuable source of human tissues/organs for the development of highly biocompatible grafts [26]. In fact, previous research demonstrated the successful development of several biological scaffolds derived from decellularized human skeletal muscle [4], omentum [61], and small-diameter vessels [62], collected as part of the aforementioned Body Donation Program.

In this work, human diaphragm harvested from cadavers was considered as an ideal, but still little investigated, source for the development of decellularized biological scaffolds for VML treatment. To the best of our knowledge, only 13 works in the literature have reported the decellularization of the diaphragmatic muscle [27,28,29,30,31,32,34,39,40,41,42,43,44], plus 5 research articles that considered the diaphragm together with other tissues for methodological studies about decellularization procedures or analysis techniques validating acellular ECM quality [33,35,36,37,38] (Table S1).

Mouse diaphragm was successfully decellularized by a detergent-enzymatic method based on the use of SDC and DNase-I for the removal of immunogenic components. This protocol allowed viable and functional scaffolds to be obtained, with high biocompatibility, pro-angiogenic, and pro-regenerative potential, which demonstrated to be re-innervated after implantation into a GFP+ Schwann cell mouse model [27,28,29,30]. The same detergent-enzymatic treatment proved to be effective on producing acellular rat diaphragm [31], which was also efficiently decellularized by muscle perfusion via vena cava [32,37,38], or immersion [33,36] in solutions of SDS, DNase-I, and ethylenediaminetetraacetic acid (EDTA). Another documented protocol for rat diaphragm decellularization was based on the use of SDS and PBS washes [34], while Sesli and collaborators [35] demonstrated that the use of SDS+Triton X-100 produced cytocompatible acellular scaffolds in comparison with freezing/thawing method plus immersion in sodium chloride (NaCl), which caused severe tissue damage. For the obtainment of acellular pig diaphragm, immersion in tri(n-butyl) phosphate (TnBP), H_2_O, and ethyl alcohol resulted in optimal tissue decellularization, ensuring scaffold cytocompatibility, but significantly affecting the biomechanical strength of the construct [41,42]. Interestingly, Boso and colleagues [44] reported the effective decellularization of pig diaphragmatic muscle by SDC and DNase-I and subsequent production of acellular ECM-derived hydrogels with mechanical stability and highly porous texture for nutrients and gas diffusion.

Similar to this work, different detergent (i.e., Triton X-100, Tween 20, SDS, SDC, and TnBP) and trypsin solutions plus RNase A and DNase-I treatment were compared for the decellularization of bovine diaphragm, demonstrating that most effective cell/DNA removal and obtainment of cytocompatible scaffolds were achieved by the use of SDS [39,40].

Only one work has so far reported the decellularization of human hemidiaphragm [44] and the use of the decellularized graft to replace the native diaphragm in a canine model, showing that the transplanted patch was completely substituted by fibrous tissue and well-integrated with the native diaphragm. Patches also appeared to be re-vascularised with peritonealisation on the abdominal site [44]. Despite pioneer collection of pre-clinical evidence, this work lacks a detailed characterisation of the human acellular diaphragmatic scaffold in terms of molecular composition, biomechanical behaviour, and propensity to cell–matrix interactions.

Although satisfying results have been reported by both in vitro and in vivo studies about the preparation of acellular diaphragmatic grafts of non-human origin, working on human-derived tissues still remains of paramount importance. Xenogeneic tissues from animals may carry residual immunogenicity due to the persistence of Galalpha1-3Galbeta1-4GlcNAc-R (alpha-gal) epitopes on the graft, which are known to elicit immune responses in xenograft recipient [63,64]. Moreover, xenogenic material may be contaminated with biological agents. Besides these issues, using decellularized grafts from the same species of the recipient assures for better matching with the histomorphology and biomolecular composition of the target tissue to be repaired [26].

Given poor experimental evidence on acellular human diaphragmatic grafts development, this work focused on comparing different protocols for the decellularization of human diaphragmatic patches. In particular, we took as a reference a previously standardized protocol that has achieved successful results on human rectus abdominis and tibialis muscles [4]; although, we needed to replace the detergent Triton X-100. This reagent, due to its potential toxicity to the endocrine human system, was recently included by the European Chemicals Agency (ECHA) in the list of substances of very high concern of the Registration, Evaluation, Authorisation and Restriction of Chemicals (REACH) Regulation. According to this, Triton X-100 is no longer usable for the preparation of products for translational purposes. Thus, we tested and compared SDS, Tergitol^TM^ and SDC as possible substitute detergents for the removal of tissue immunogenic components within human diaphragm. The use of detergents represents one of the most common methods to obtain ECM biologic scaffolds, since they are capable to solubilise cell membranes and dissociate DNA from proteins. Chemical detergents for decellularization may be non-ionic reagents, such as Tergitol^TM^, which act by disrupting DNA–protein, lipid–lipid, and lipid–protein interactions, helping to maintain native protein structures. Conversely, ionic surfactants such as SDS and SDC completely solubilise cell and nucleic membranes, with a major probability of protein denaturation [65,66,67]. In this study, both categories of detergents were tested, also investigating a combination of the two in order to mitigate the effects of the ionic detergent SDS with the subsequent use of the non-ionic reagent Tergitol^TM^. Before detergent treatment, tissue immersion in deionised water helped cell lysis by osmotic shock, while the exposure to enzymes such as DNase-I and trypsin enhanced cell removal and the complete degradation of nuclear components from the tissue [68].

In particular, DNase-I promoted the cleavage of nucleic acid sequences and the depletion of nucleotides, whereas trypsin disrupted cell–cell and cell–matrix bonds into the tissue, being used in a sensitive-time manner and in combination with the chelating agent EDTA, to prevent eventual damage to collagen/elastic fibres [69]. Remarkably, each multiple-step protocol was performed only one time, achieving efficient decellularization in 6 days of treatment and, thus, highly reducing the time—and costs—for graft fabrication.

Together with the removal of cellular antigens, which induces an adverse immune response by the host, the preservation of ECM is crucial to provide tissue-specific molecular and mechanical stimuli that contribute to cellular homeostasis, sustain wound healing and tissue repair, as well as assuring for functional recovery [67]. The skeletal muscle ECM is a complex meshwork of collagens, glycoproteins, proteoglycans, and elastin [70], which must be adequately preserved even after treatment with chemical detergents and enzymes, so that a bioactive scaffold is obtained. Histological investigations on decellularized diaphragmatic sections confirmed the effectiveness of the different protocols in terms of cell removal, as well as myofibril depletion. According to the existing literature on diaphragm decellularization, both the preservation [27,29,38,44] and the total removal [32,35] of muscle fibres were reported, with their complete depletion lowering the risk of immunogenic reactions.

Besides the removal of immunogenic elements, the preservation of the muscular ECM composition and three-dimensional (3D) organisation also needs to be assessed after decellularization. Within the skeletal muscle ECM, Collagen I fibres are present in the endo-, peri-, and epimysium, assuming to confer tensile strength and stiffness to the tissue [70]. As shown by the localisation of the specific labelling around muscle fibres, Collagen IV and Laminin are the major components of the specialised basement membrane found at the interface between the endomysium and the myofiber sarcolemma [70]. The preservation of these proteins after decellularization is of paramount importance, since their depletion can negatively affect cell–matrix interactions, altering repopulating cell adhesion, proliferation, survival, and behaviour [71]. Along with positive markers, negative expression of major histocompatibility complex (MHC) molecules may provide some insight into tissue immunogenicity. Being responsible to elicit adverse host immune response in vivo, the expression of the class I and II major histocompatibility complexes (MHC I and II) was previously proved to be absent after rat diaphragm decellularization [31,32]. Accordingly, this study demonstrated that decellularized diaphragmatic patches did not express the MHC class II cell surface receptor HLA-DR, which supports their low immunogenicity. Additionally, the evaluation of glycosaminoglycans appears to be relevant if we consider that these polysaccharides actively contribute to the ECM role in regulating the pool of satellite cells. Specifically, GAGs and other proteoglycans have been shown to promote satellite cell differentiation and fusion into mature myofibers [70]. Being based on the use of a non-ionic detergent alone, Protocol n. 4 determined the lowest depletion of GAGs, in agreement with reports about less harmful effect of this type of reagents towards these molecules [67].

For the first time in our knowledge, collagen imaging by SHG microscopy was performed to characterise the acellular diaphragmatic ECM. SHG imaging has been frequently used to assess the quality of the fibrillar collagen in the normal versus the disease state of different tissues [72]. However, this technology is also particularly useful in decellularization–recellularization studies, to discriminate between the collagen/elastin of the scaffolds and the original cells or the recellularizing tissue [73]. Reported evidence on SHG imaging of decellularized versus native tissues detected a significant decrease in signal intensity from the orderly packed collagen fibres of native samples after detergent-enzymatic treatment. This signal reduction is associated with structural damage to collagen fibres due to decellularization [74,75]. Interestingly, the SHG analysis performed in this work showed that the decellularization treatment did not depauperate the collagen fraction, in accordance with morphometric quantification. Thus, we can infer that no disrupting effects on the collagen structure were exerted by the tested detergents, whereas local orientation of collagen fibres was preserved only by Protocol n. 2, using both SDS and Tergitol^TM^.

Following the characterisation of ECM structure and composition, the analysis of the mechanical behaviour of acellular tissue patches is fundamental to ensure suitable mechanical performances in vivo. Uniaxial tensile tests allowed tissue stiffness and anisotropy in a physiological strain range for skeletal muscle to be evaluated. The comparison of secant elastic modulus among native and decellularized diaphragmatic tissue showed a decrease in the anisotropy degree after decellularization. This is consistent with the SHG analysis, where a random fibre orientation was detected in decellularized samples. Significant differences in stiffness were found among samples obtained with different protocols. A lower stiffness was measured following Protocols n. 1, n. 2, and n. 3, which may be ascribable to a slight reduction in Collagen I fibres, which normally occurs after decellularization. Differently, in the case of diaphragmatic patches treated with Protocol n. 4, a higher stiffness value was found both in transversal and longitudinal direction. At the same time, at the first glance these samples showed an altered consistency and a slight size increase after decellularization with Tergitol^TM^ detergent. Based on these findings, Protocol n. 4 may be considered able to trigger tissue stretching with an associated reduction in the crimping of collagen fibres. In this case, the decellularized tissue would behave as a pre-stretched tissue, showing a stiffer mechanical behaviour. These modifications in anisotropy and stiffness may be further investigated by extending the number of subjects and samples. However, it should be considered that the optimal stiffness of a decellularized patch depends on the mechanical properties of the specific skeletal muscle to be repaired. Therefore, each of these protocols cannot be excluded a priori.

The biocompatibility assessment of bio-engineered scaffolds plays an essential role to provide evidence about the appropriate biological response and inertness before implant into the patient [76]. In vitro safety testing of acellular grafts may be accomplished by cytotoxicity tests, which verify the eventual remnants of harmfulness chemical detergents and reagents within the decellularized tissue, as well as the preservation of the ECM biochemical cues, which can favour cell adhesion and proliferation. Herein, the in vitro cytotoxicity assay by indirect co-culture of Ad-MSCs with acellular diaphragmatic patches highlighted the biological safety of the scaffolds, setting the stage for graft studies into animal models. As a further step, cell–matrix interactions were investigated by seeding Ad-MSCs onto the decellularized scaffolds, which were effectively colonised by a cell monolayer, regardless of the detergent-enzymatic protocol used. Corroborating characterization data on muscular ECM quality, the successful cell growth on acellular diaphragmatic scaffolds demonstrated that they were prepared as bioactive substrates preserving a complex of biomolecular cues that support cell viability and growth.

In vivo biocompatibility by subcutaneous implant into Balb/c mice was investigated for diaphragmatic tissue decellularized by Protocol n. 2. In fact, even if the tested detergents demonstrated that they were equivalent in efficiently depleting immunogenic material and preserving diaphragmatic ECM, the combination of SDS and Tergitol^TM^ seemed to offer the advantages of both ionic and non-ionic decellularization reagents. Mainly based on SHG data for collagen preservation, slightly better decellularization results seemed to be achieved by Protocol n. 2, which led to a significantly higher preservation of collagen fibre organisation/orientation.

Failure to effectively decellularize a tissue causes adverse outcomes upon in vivo implantation, eliciting a pro-inflammatory response, which involves macrophages recruitment and leads to fibrosis. This reaction to graft material can cause seroma and sterile abscess formation, as well as chronic inflammation, which may lead to graft rejection [77]. Thus, subcutaneous implantation represents an important step in validating the quality of decellularized scaffolds. Besides determining in vivo, the efficient removal of immunogenic cell material, this investigation allows the assessment of local tissue response to aggressive chemical reagents used during decellularization and eventually persisting in complexes with the ECM proteins [36]. For this reason, the non-immunogenicity of mouse and rat decellularized diaphragm was previously verified by in vivo subcutaneous implantation studies [28,32,36]. Our pre-clinical evaluation of tissue reaction to human decellularized diaphragmatic disks highlighted the presence of a moderate lympho-monocytic infiltrate at the graft–host interface, which is the result of the innate animal response to muscular matrix scaffolds. Indeed, the surgical introduction of a foreign material into living host tissue firstly leads to an acute inflammatory reaction, together with other events, i.e., the formation of granulation tissue and fibrosis or fibrous encapsulation of the graft [78]. The acute inflammation phase involves the infiltration of inflammatory cells such as lymphocytes, which exhibit a cytokine secretory activity being able to recruit monocytes/macrophage to the site of injury [78,79]. These cells are known to be responsible for graft degradation in vivo, possibly defining the overall remodelling outcome of the implanted device [80]. Besides providing evidence of graft biocompatibility, in vivo studies showed that an early angiogenetic process was elicited, being supported by the infiltration of VEGF-positive cells. These results paved the way to carry out in vivo transplantation of human acellular diaphragmatic scaffolds into the damaged muscle of VML models. This experimental setting will allow the assessment of the capability of bio-engineered diaphragmatic scaffolds to be revascularized and reinnervated, which is essential for the development of bioactive and functional grafts.

## 5. Conclusions

Despite recent progress in biomaterials fabrication for VML regenerative therapies, there is still an unmet need for scaffolds that highly resemble the characteristics of human skeletal muscle ECM to promote functional tissue recovery. Generation of ECM scaffolds through detergent-enzymatic decellularization treatments proved to remove immunogenic cellular and nuclear components while maintaining biological activity, mechanical integrity and 3D structure of the native tissue. In this work, the human decellularized diaphragm was shown to be a promising candidate for the development of biological scaffolds intended for skeletal muscle regeneration in VML patients. Ionic and non-ionic detergents were compared for tissue decellularization, with no clear superiority of one over the others. Nevertheless, tissue manipulability and mechanical integrity seemed to be more compromised by long incubation with Tergitol^TM^, while the combination of ionic (SDS) and non-ionic (Tergitol^TM^) detergents appeared to produce the most favourable results in terms of collagen preservation. Based on that, Protocol n. 2 was considered to have achieved slightly better decellularization outcomes, revealing high biocompatibility and angiogenetic effect in vivo. In this work, fine pre-clinical characterisation of the acellular human diaphragmatic patches was performed for the first time and contributed to a better understanding of the effect of the cell-removing process on ECM, as well as to further develop diaphragm decellularization technology. Just as relevantly, this study demonstrated the fabrication of bio-safe allografts that showed no toxicity towards cell cultures and no immunogenicity once subcutaneously implanted into mice models. Interestingly, the implant of acellular diaphragmatic grafts into animal models of VML injury can also be carried out to demonstrate functional performance of the scaffolds.

## Figures and Tables

**Table 1:** Overview of the decellularization protocols tested for human diaphragmatic patches.

Protocol n.	1	2	3	4
Method	dH_2_O	dH_2_O	dH_2_O	dH_2_O
	(24 h at 4 °C)	(24 h at 4 °C)	(24 h at 4 °C)	(24 h at 4 °C)
	DNase in NaCl 1 M	DNase in NaCl 1 M	DNase in NaCl 1 M	DNase in NaCl 1 M
	(3 h at RT)	(3 h at RT)	(3 h at RT)	(3 h at RT)
	0.05% Trypsin +	0.05% Trypsin +	0.05% Trypsin +	0.05% Trypsin +
	0.02% EDTA in PBS	0.02% EDTA in PBS	0.02% EDTA in PBS	0.02% EDTA in PBS
	(1 h at 37 °C)	(1 h at 37 °C)	(1 h at 37 °C)	(1 h at 37 °C)
	0.5% SDS +	0.5% SDS +	0.5% SDC +	0.5% Tergitol^TM^ +
	0.8% NH_4_OH in PBS	0.8% NH_4_OH in PBS	0.8% NH_4_OH in PBS	0.8% NH_4_OH in PBS
	(72 h at 4 °C)	(48 h at 4 °C)	(72 h at 4 °C)	(72 h at 4 °C)
		0.5% Tergitol^TM^ +		
		0.8% NH_4_OH in PBS		
		(24 h at 4 °C)		
	dH_2_O	dH_2_O	dH_2_O	dH_2_O
	(48 h at 4 °C)	(48 h at 4 °C)	(48 h at 4 °C)	(48 h at 4 °C)

## Supplementary Materials

The following are available online at https://www.mdpi.com/article/10.3390/biomedicines10040739/s1, Table S1: Current literature on the decellularization of diaphragmatic muscle for tissue engineering applications.
