# Supplementary material for: Preclinical Development of Bioengineered Allografts Derived from Decellularized Human Diaphragm
Source: Biomedicines. 2022 Mar 22;10(4):739. doi: 10.3390/biomedicines10040739 (PMC9031975; doi:10.3390/biomedicines10040739)
Supplement: Supplementary file 1 [file biomedicines-10-00739-s001.zip › biomedicines-1514243-supplementary.pdf]

Supplementary Materials

Table S1. Current literature on the decellularization of diaphragmatic muscle for tissue engineering applications.

Reference	Species	Clinical Target	Decellularization Method	Investigations on Acellular ECM	Major Findings
Conconi et al., 2009 [31]	Rat	CDH	Immersion in:<br>- H_2O for 72 h at 4 °C<br>- 4% SDC for 4 h<br>- 2000 kU DNase-I in 1 M NaCl for 3 h<br>*2 DET cycles*	Characterization studies<br>- IHC analysis for the localization of MHC class I and II antigens, bFGF and TGF-β<br>- CAM assay<br>In vitro<br>- Seeding of myoblasts on acellular matrices<br>In vivo<br>Implant of acellular diaphragmatic matrices ± autologous myoblasts or/and VEGF-loaded porous silica gel to repair a surgically created defect in the rat diaphragm	- Complete removal of nuclear elements<br>- Negative expression of MHC class I and II antigens<br>- Strong and diffuse immunoreactivity against bFGF and TGF-β<br>- Strong angiogenic response exerted by decellularized diaphragmatic matrix on CAM<br>- Myoblast adhesion, proliferation and fusion onto the acellular matrices<br>- Low immunoreactivity, muscle fibers neo-formation, vascularization and cholinergic fiber detection within implants composed of acellular diaphragmatic matrix and myoblasts
Cozad et al., 2011 [41]	Pig	Wound healing	Immersion in:<br>- 1% (v/v) TnBP solution for 48 h at RT, under continuous agitation<br>- H_2O for 24 h at RT, under continuous agitation<br>- 70% ethyl alcohol for 24 h at RT, under continuous agitation	Characterization studies<br>- Cross-linking of decellularized diaphragmatic disks with AuNR and AuNPs<br>- SEM analysis<br>- Free radical scavenging study on composite scaffolds by ROS assay<br>In vitro<br>- Seeding of L-929 mouse fibroblast cells on decellularized and composite scaffolds	- Ability to sustain fibroblast adhesion and proliferation by both decellularized and composite scaffolds<br>- Modification of free radical generation according to nanomaterial shape and concentration
Davari et al., 2016 [44]	Human	Diaphragm regeneration	Immersion in:<br>- H_2O for 48 h at 4 °C<br>- 4% SDC for 4 h<br>- 2000 kU DNase-I in 1 M NaCl for 4 h<br>*25 DET cycles*	Characterization studies<br>Hematoxylin and eosin staining of decellularized *versus* native tissue sections<br>In vivo<br>Replacement of the native diaphragm with 10 × 7 cm patches of cryopreserved or decellularized heterograft in a dog model of diaphragmatic damage	- Scattered fibrotic bands and mild to moderate elevation of the left hemidiaphragm showed by CT scan in operated muscles<br>- No evidence of gross disruption and complete healing of the suture line<br>- Complete replacement of implanted patches by dense fibrous tissue<br>- Less inflammatory cell infiltration and scattered foreign body granulomas in decellularized versus cryopreserved patch grafts
Gubareva et al., 2016 [32]	Rat	Diaphragm regeneration	Perfusion via vena cava with:<br>- 4% (w/v) SDC for 3 h<br>- PBS for 10 min<br>- 2000 ku DNase-I<br>- 2 mM EDTA in H_2O, 2 washes of 30 min each<br>- PBS for 12 h<br>*All steps were performed under agitation at RT*	Characterization studies<br>- DNA quantification<br>- Histological evaluation<br>- SEM analysis<br>- IHC study of ECM markers (i.e., laminin, elastin, collagen I and IV, cytokeratins)<br>- IF study of MHC I and II antigens, tropomyosin, VEGF, vWF<br>- Mechanical tests<br>- Pro- and antioxidant assay by EPR<br>In vitro	- Preservation of tissue architecture and mechanical properties in the decellularized diaphragmatic scaffolds<br>- BM-MSC adhesion, proliferation and differentiation supported by the acellular diaphragmatic matrix<br>- High biocompatibility of acellular grafts demonstrated by subcutaneous implant study<br>- Myography, spirometry parameters, and histological evaluations of damaged and grafted diaphragms similar to native controls<br>- In situ regeneration of functional tissue observed into diaphragm injury models grafted with reseeded acellular scaffolds

				- Studies on adhesion, proliferation and differentiation of rat BM-MSCs seeded on scaffolds<br>In vivo<br>- Subcutaneous implant<br>- Orthotopic transplantation into a rat model of diaphragmatic damage	
Gubareva et al., 2016 [33]	Rat	-	Immersion in:<br>- H_2O<br>- 4% (w/v) SDC<br>- PBS<br>- DNase-I<br>- EDTA<br>*All steps were performed under rotary agitation*	Characterization studies<br>ERP spectroscopy to determine the concentrations of paramagnetic centers, which reflect the vital functions of cellular structures, into decellularized *versus* native tissue	- Paramagnetic centers with g-factor = 2.007 at a concentration of 10^{-8} mol/g of lyophilized tissue detected into the native diaphragm, demonstrating the presence of living cellular elements in the sample<br>- No identification of paramagnetic centers into the decellularized diaphragm, revealing that it does not contain a system of electron carriers, required for the functioning of living cells
Piccoli et al., 2016 [27]	Mouse	Diaphragm regeneration	Immersion in:<br>- H_2O for 24 h at 4 °C<br>- 4% SDC for 4 h<br>- 2000 kU DNase-I in 1 M NaCl<br>for 3 h<br>*3 DET cycles*	Characterization studies<br>- Histological evaluations<br>- DNA, GAGs, collagen and elastin quantification<br>- SEM analysis<br>- Mechanical tests<br>In vivo<br>- Implant of decellularized patches over a non-injured mouse diaphragm<br>- Implant of decellularized patches into an atrophic mouse diaphragm	Generation of a positive local immunoresponse, activation of a pro-regenerative environment and stimulation of host muscle progenitor cell activation/migration were observed in both wild-type and pathological mouse models grafted with decellularized diaphragmatic ECM
Smith et al., 2016 [42]	Pig	ACL reconstruction	Immersion in:<br>- 1% (v/v) TnBP solution for 24 h at RT<br>- H_2O for 48 h at RT<br>- 70% ethyl alcohol for 24 h at RT	Characterization studies<br>- Conjugation of AuNPs and nano-HAp to decellularized diaphragmatic tissue<br>- TEM analysis<br>- Differential scanning calorimetry<br>In vitro<br>- Seeding of L-929 mouse fibroblasts on decellularized and composite scaffolds	- Decreased cytocompatibility of decellularized diaphragmatic scaffolds added with < 200 nm nano-Hap<br>- Enhanced cytocompatibility assured by the addition of AuNPs to decellularized diaphragmatic scaffolds, also in the presence of <200 nm nano-Hap<br>- Increased cytocompatibility of scaffolds added with < 40 nm nano-HAp compared to scaffolds crosslinked without nanoparticles<br>- Improvement of decellularized diaphragmatic ECM properties by addition of AuNPs and < 40nm nano-HAp
Liao et al., 2017 [34]	Rat	CDH	Immersion in:<br>- 0.5% SDS for 48 h under rotation<br>- PBS for 24 h under rotation	Characterization studies<br>- Mechanical tests<br>- TEM analysis<br>In vitro<br>- Seeding of hAFMSCs on decellularized scaffold by infusion system<br>In vivo<br>- Orthotopic implant of decellularized scaffolds ± hAFMSCs for the surgical repair of diaphragmatic defect in a rat model	- Improved physiological function of diaphragmatic scaffolds loaded with hAFMSCs<br>- Ability of the repopulated grafts to accelerate the functional recovery of diaphragmatic hernia

Alvarèz Fallas et al., 2018 [28]	Mouse	Diaphragm regeneration	Immersion in:<br>- H_2O for 24 h at 4 °C<br>- 4% SDC for 4 h<br>- 2000 kU DNase-I in 1 M NaCl for 3 h<br>*3 DET cycles*	<u>Characterization studies</u><br>- CAM assay<br>- Proteome Profiler Angiogenesis Array<br>- ELISA for detection of VEGF, HGF, EGF and SDF-1a into the scaffold<br><u>In vitro</u><br>- Seeding of HUVECs on decellularized scaffolds<br><u>In vivo</u><br>- Subcutaneous implant in a mouse model to evaluate angiogenic potential of the acellular scaffold<br>-Orthotopic implantation of the acellular scaffold into murine host diaphragm without any local injury, to evaluate angiogenic properties of the graft	- Detection of pro-angiogenic molecules in the decellularized tissue by CAM assay and protein array<br>- Vascularization of the ECM-derived scaffold 7 days post subcutaneous implantation<br>- Detection of newly formed blood vessels containing CD31-, αSMA-, and vWF-positive cells detected inside the scaffold after orthotopic implant
Sesli et al., 2018 [35]	Rat	-	**METHOD A**<br>Immersion in:<br>- 0.5% SDS for 48 h<br>- 1% SDS for 24 h<br>- Washes with PBS<br>- 1% TritonX-100 solution for 1 h.<br>*Decellularization steps were carried out at RT.*<br><br>**METHOD B**<br>Freezing at -80°C for 30 min and thawing at RT for 15 min (3 cycles)<br>Immersion in:<br>- 0.5 M NaCl for 4 h<br>- 1 M NaCl for 4 h<br>- H_2O overnight<br>- 0.25% trypsin/EDTA for 2 h<br>- H_2O<br>*After freezing-thawing, other steps were performed with 120 rpm agitation at RT*	<u>Characterization studies</u><br>- Histological evaluations<br><u>In vitro</u><br>- Seeding of AdMSCs on lyophilized acellular scaffolds	- Efficient decellularization and high cytocompatibility of diaphragmatic scaffolds prepared by Method A<br>- Severe tissue damage in diaphragm caused by Method B
Sotnichenko et al., 2018 [36]	Rat	-	Immersion in:<br>- H_2O<br>- 4% (w/v) SDC<br>- PBS<br>- DNase-I<br>- EDTA<br>*All steps were performed under rotary agitation*	<u>In vivo</u><br>Subcutaneous implantation of decellularized diaphragmatic matrix in the interscapular region of rat models	**Postoperative day 7:**<br>- Formation of a granulation tissue capsule (up to 900 μm thick) around the matrix and growth of fibroblast fibers into the implant<br>- Intensive inflammatory reaction, with the infiltrate containing mononuclear cells (T and B lymphocytes and macrophages) and segmented leukocytes, including eosinophils<br>- In tissues surrounding the capsule, detection of pronounced edema and plethoric blood vessels, especially around the implant<br>**Postoperative day 14:**<br>- Detection of less pronounced edema

Gubareva et al., 2019 [37]	Rat	-	Perfusion via vena cava with:<br>- 4% (w/v) SDC for 3 h<br>- PBS for 10 min<br>- 2000 ku DNase-I<br>- 2 mM EDTA in H_2O, 2 washes of 30 min each<br>- PBS for 12 h<br>*All steps were performed under agitation at RT*	<u>Characterization studies</u><br>-IHC for the visualization of ECM proteins (i.e., elastin, collagen IV, collagen I, laminin and fibronectin)<br>- Free-radical oxidation study by chemiluminescence method to assess the degree of ECM decellularization and the viability of cellular structures in recellularized diaphragmatic tissue	- Compaction, degradation and resorption by macrophages of the diaphragmatic ECM<br>- Growth of newly formed vessels and productive capillaritis<br>- Considerable decrease of the inflammatory reaction both in the sample and in surrounding tissues, due to lower number of activated macrophages and T cells<br>- No pronounced qualitative changes in decellularized ECM structure (data not shown)<br>- Significant difference detected among the chemiluminescence curves of the native and recellularized diaphragm tissues and the chemiluminescence curves of the same muscle tissues after decellularization<br>- Possibility to use the method to discriminate the presence of viable cells within the native, decellularized and recellularized tissues
Morokov et al., 2019 [38]	Rat	-	Perfusion via vena cava with:<br>- 4% (w/v) SDC for 3 h<br>- PBS for 10 min<br>- 2000 ku DNase-I<br>- 2 mM EDTA in H_2O, 2 washes of 30 min each<br>- PBS for 12 h<br>*All steps were performed under agitation at RT*	<u>Characterization studies</u><br>- Histological evaluation by hematoxylin and eosin staining<br>- Sample collagenization and ultrasound investigation by high-frequency acoustic microscopy for noninvasive evaluation and visualization of volume microstructure of the diaphragmatic ECM	- Preservation of the orientated structure of the muscle fibers and absence of the cell content showed in acellular diaphragm by histological analysis<br>- Preservation of the integral architecture of the ECM after the decellularization demonstrated by the acoustic imaging
Trevisan et al., 2019 [29]	Mouse	CDH	Immersion in:<br>- H_2O for 24 h at 4 °C<br>- 4% SDC for 4 h<br>- 2000 kU DNase-I in 1 M NaCl<br>for 3 h<br>*3 DET cycles*	<u>Characterization studies</u><br>- Testing of different conditions for ECM storage<br>- Collagen, sGAG and elastin quantification<br>- Histological evaluations<br><u>In vitro</u><br>- Seeding of human pediatric MPCs on acellular scaffolds<br>- Exposure of the engineered diaphragmatic construct to cardiotoxin injury	- Development and characterization of a storable and ready-to-use acellular diaphragmatic ECM<br>- Preservation of the structure and architecture of decellularized ECM after storage<br>- Acellular scaffold ability to activate a regenerative response in vitro promoting cell self-renewal and a positive ECM remodeling<br>- Generation of a viable and functional construct by the combination of animal-derived ECM and human MPCs
Trevisan et al., 2019 [30]	Mouse	CDH	Immersion in:<br>- H_2O for 24 h at 4 °C<br>- 4% SDC for 4 h<br>- 2000 kU DNase-I in 1 M NaCl for 3 h<br>*3 DET cycles*	<u>In vivo</u><br>- Orthotopic implant of acellular diaphragmatic patches into a CHD mouse model<br>- Implantation into a GFP+ Schwann cell mouse model to investigate scaffold reinnervation	- Absence of rejection or hernia recurrence after implant<br>- Generation of new blood vessels, promotion of long-term muscle regeneration, recovering of host diaphragmatic function after diaphragm-derived ECM grafting<br>- Reinnervation of implanted diaphragmatic patches
Boso et al., 2020 [43]	Pig	CDH	Immersion in:<br>- H_2O for 24 h at 4 °C<br>- 4% SDC for 4 h<br>- 2000 kU DNase-I in 1 M NaCl<br>for 3 h<br>*4 DET cycles*	<u>Characterization studies</u><br>- DNA quantification<br>- Proteomic analysis by Mass Spectrometry<br>- Decellularized ECM-derived hydrogel preparation<br>- SEM analysis<br>- FRAP measurements<br>- IF for the localization of ECM proteins (i.e., laminin. Collagen I and IV)<br>- Collagen and hyaluronic acid quantification	- Adequate preservation of ECM proteins in the decellularized diaphragmatic scaffold<br>- Obtainment of diaphragmatic dECM-derived hydrogels with mechanical stability and an intricate inner ultrastructure, with highly porous texture for nutrients and gas diffusion<br>- Demonstration of hydrogel biodegradation susceptibility and cytocompatibility<br>- No liver herniation, good strength capability and hydrogel repopulation by resident cells after implant in the CHD murine model

				- Rheometric analysis<br>- Gelation kinetics study<br>- Enzymatic degradation assay<br>In vitro<br>- Co-seeding of hSKMCs and hFbs onto acellular diaphragmatic hydrogel coatings<br>- Ex vivo CHD model<br>In vivo<br>- Implantation of cross-linked acellular diaphragmatic hydrogels as stand-alone patches into a CHD mouse model	
Vellachi et al., 2020 [39]	Bovine	Abdominal wall repair	Immersion in:<br>- 0.5% solution of Triton X-100/Tween 20/SDS/SDC/tri(n-butyl)phosphate/ trypsin for 12 h or 24 h<br>- 0.2 mg/ml RNaseA and 0.2 mg/ml DNase I for 24 h<br>- PBS for 24 h<br>*All steps were performed in an orbital shaker at 37°C*	Characterization studies<br>- DNA quantification<br>- Histological evaluation by hematoxylin and eosin staining<br>- Hydration analysis<br>- Matrix degradation analysis<br>- Mechanical tests<br>- Cytotoxicity extract test on r-BMSc	- Optimum decellularization achieved with 0.5% SDS for 12 h<br>- High water absorption capacity and low rate of matrix degradation<br>- Significant reduction in tissue biomechanical strength observed following decellularization<br>- No cytotoxicity imparted by ECM extracts for the proliferation of r-BMSc
Vellachi et al., 2020 [40]	Bovine	Abdominal wall repair	Immersion in:<br>- 0.5% SDS for 12 h<br>- 0.2 mg/ml RNaseA and 0.2 mg/ml DNase I for 24 h<br>- PBS for 24 h<br>*All steps were performed in an orbital shaker at 37°C*	In vitro<br>Seeding of r-BMSc on decellularized diaphragm<br>In vivo<br>Repair of full-thickness abdominal wall defects in a rabbit model by using acellular diaphragmatic ECM ± r-BMSc	- Promotion of muscle regeneration in terms of collagen deposition, maturation, neovascularization, and lack of any significant adhesions with the abdominal viscera after implant of decellularized scaffolds<br>- Significantly increased collagen deposition and biomechanical strength of the scaffolds seeded with r-BMSc<br>- In case of scaffolds seeded with r-BMSc, significant reduction in antibody and cell mediated immune reactions to the xenogeneic tissue in rabbit model.

Abbreviations: ACL, anterior cruciate ligament; AdMSCs, adipose derived mesenchymal stem cells; AuNPs: gold nanoparticles; AuNR, gold nanorod; bFGF, basic Fibroblast Growth Factor; BM-MSCs, bone marrow mesenchymal stem cells; CAM, chorio-allantoic membrane; CDH, congenital diaphragmatic hernia; CT, computed tomography; dECM, decellularized extracellular matrix; DET, detergent-enzymatic treatment; ECM, extracellular matrix; EDTA: ethylenediaminetetraacetic acid; EGF, Epidermal Growth Factor; EPR, electron paramagnetic resonance; FRAP, fluorescence recovery after photobleaching; GFP, Green Fluorescent Protein; hAFMSC, human amniotic fluid-derived multipotent stromal cells; HAp, hydroxyapatite; hFbs, human dermal fibroblasts; HGF, Hepatocyte Growth Factor; hSKMCs, human Skeletal Muscle Cells; HUVECs, Human Umbilical Vein Endothelial Cells; IF, immunofluorescence; IHC, immunohistochemistry; MPCs, Muscle Progenitor Cells; NaCl, sodium chloride; PBS, phosphate buffer saline; r-BMSc, rabbit bone marrow-derived mesenchymal stem cells; ROS, reactive oxygen species; RT, room temperature; SDC, sodium deoxycholate; SDF-1a, stromal cell-derived factor-1a; SEM, transmission electron microscopy; sGAG, sulfated glycosaminoglycan; TEM, transmission electron microscopy; TGF-β, Transforming Growth Factor-β; TnBP, tri(n-butyl) phosphate; VEGF, Vascular Endothelial Growth Factor; vWF, von Willebrand Factor; αSMA, Alpha Smooth Muscle Actin.
